# *Clostridium Perfringens* Enterotoxin (CPE) and CPE-Binding Domain (*c*-CPE) for the Detection and Treatment of Gynecologic Cancers

**DOI:** 10.3390/toxins7041116

**Published:** 2015-04-01

**Authors:** Jonathan D. Black, Salvatore Lopez, Emiliano Cocco, Carlton L. Schwab, Diana P. English, Alessandro D. Santin

**Affiliations:** 1 Department of Obstetrics, Gynecology and Reproductive Sciences, Yale University School of Medicine, 333 Cedar Street, PO Box 208063, New Haven, CT 06520-8063, USA; E-Mails: jonathan.black@yale.edu (J.D.K.); emiliano.cocco@yale.edu (E.C.); carlton.schwab@yale.edu (C.L.S.); diana.english@yale.edu (D.P.E.); 2 Division of Gynecologic Oncology, University Campus Bio-Medico of Rome, Via Alvaro del Portillo 21, 00128 Rome, Italy; E-Mail: s.lopez@unicampus.it

**Keywords:** clostridium perfringens enterotoxin, gynecologic cancer, Claudin, therapeutics, diagnostics

## Abstract

*Clostridium perfringens* enterotoxin (CPE) is a three-domain polypeptide, which binds to Claudin-3 and Claudin-4 with high affinity. Because these receptors are highly differentially expressed in many human tumors, claudin-3 and claudin-4 may provide an efficient molecular tool to specifically identify and target biologically aggressive human cancer cells for CPE-specific binding and cytolysis. In this review we will discuss these surface proteins as targets for the detection and treatment of chemotherapy-resistant gynecologic malignancies overexpressing claudin-3 and -4 using CPE-based theranostic agents. We will also discuss the use of fluorescent *c*-CPE peptide in the operative setting for real time detection of micro-metastatic tumors during surgery and review the potential role of CPE in other medical applications.

## 1. Introduction

*Clostridium perfringens* enterotoxin (CPE) is a single polypeptide of 35 kDa. It consists of three domains; (1) *C*-terminal domain I, which is responsible for receptor binding; (2) domain II, which is responsible for oligomerization and membrane insertion; and (3) domain III, which may participate in physical changes when the CPE protein inserts into membranes. CPE is comprised of 319 amino acids. It is the virulence factor responsible for *Clostridium perfringens* type A food poisoning, which is the second most commonly reported food-borne illness in the United States [1]. The structure and function of CPE has been investigated through the characterization of the functional properties of enterotoxin fragments and point mutations [2,3,4,5,6]. The CPE_290–319_ COOH-terminus fragment is adequate for high-affinity binding to target cell receptors. However, this fragment is not capable of initiating large complex formation or causing cytolysis [7]. Of note, the CPE_290-319_ COOH-terminus fragment inhibits cytolysis of target cells by full-length CPE [8]. Residues CPE_45-116_ are essential for large complex formation and cytotoxicity. When the NH_2_ terminus is deleted, a CPE_45-319_ fragment is generated, which facilitates enhanced large membrane complex formation and cytotoxic activity [9]. Claudin-3, claudin-4, claudin-6, claudin-8 and claudin-14 [10,11] have been shown to represent natural receptors for CPE; they are the members of the transmembrane tissue-specific claudin proteins capable of facilitating CPE binding and cytolysis [12,13]. 

## 2. Role of Novel Treatments in Chemotherapy Resistant Gynecologic Cancer

Although many patients with gynecologic malignancies may initially respond to cytoreductive surgery plus platinum/taxane chemotherapy, and/or radiation, many experience recurrences and a poor-prognosis [14,15,16]. The development of cisplatin resistance reduces therapeutic effectiveness. Resistance mechanisms are often a result of intrinsic pathway activation used during development or as a defense mechanism against environmental toxins [17]. 

Patients with platinum-resistant/refractory ovarian cancer are known to have a poor prognosis and are classified as having chemotherapy-resistant/refractory ovarian cancer [18]. In these cases, single-agent therapies are used to treat this subset of patients. These include paclitaxel, doxorubicin, and topotecan. The response rate is low, at approximately 10%–15%, and overall survival is approximately 12 months [19]. Trials of combination chemotherapy in platinum-resistant ovarian cancer have failed to improve overall survival. Notably, it increases toxicity [20]. An encouraging discovery in ovarian cancer research has been that chemotherapy-resistant ovarian tumors express claudin-3 and -4 genes at considerably higher levels when compared with chemotherapy-naive ovarian tumors [21,22]. Preclinical studies have investigated the utility of this finding and have found that chemotherapy-resistant ovarian cancer cell lines continue to display substantial sensitivity to CPE *in vitro* and *in vivo* despite their resistance to multiple different chemotherapeutic agents [23]. 

For patients with endometrial cancer who failed first line therapy, a combination regimen is the most effective. However, there is no established and universally recommended second-line agent in this disease. In patients with measurable disease, second-line agents produce a response in only 50% of patients and a complete response is infrequent. While progression free survival (PFS) and overall survival (OS) times are improving, the 5-year survival rate with advanced/recurrent measurable disease is still <10% [16]. Low initial complete response rates and the high rate of recurrence and/or progression suggest de novo and/or rapidly developing drug resistance. The underlying causes of drug resistance are multifactorial. In endometrial cancer, the resistance is mostly related to the overexpression of β-tubulin subtypes and/or the multidrug-resistance gene (*MDR-1*). Inhibition of apoptosis via alterations in the extrinsic and intrinsic apoptosis pathways and the PI3KCA pathway are also known to occur [16].

Patients with recurrent cervical cancer, which is not amenable to surgery or radiation, have a poor prognosis. The most common treatment for recurrent or metastatic cervical cancer is a combination of paclitaxel and cisplatin or paclitaxel, cisplatin and bevacizumab [24,25,26]. Although not curative, these treatments result in median survival times of 1–1.5 years. When a patient progresses after this initial therapy for a recurrence or metastases, there are no FDA approved or NCCN level 1 or 2A therapies available and therefore, options are limited [24,25,26,27].

### 2.1. Claudins

Before Mikio Furuse and Shoichiro Tsukita discovered claudins-1 and -2 in 1998, it was thought that tight junctions were mediated by occludins alone [28]. The discovery of this new class of proteins within tight junctions led to an investigation into the proteomics of tight junctions. Ultimately, this led to the discovery of additional claudins, most notably, claudins-3 and -4 [29]. Today there are 27 members of the claudin family of proteins. This family of proteins is primarily known for the role they play in mediating tight junctions between cells; they create a physiologic tight barrier, which prevents unregulated diffusion of water, lipids, proteins and anions. Many claudins (claudins-1, -3, -5, -11, -14, -19, and tricellulin) have sealing functions as well. Other claudins form channels across tight junctions which feature selectivity for cations (claudins-2, -10b, and -15), selectivity for anions (claudin-10a and -17), or are permeable to water (claudin-2). For several claudins (claudins-4, -7, -8, -16, and occludin), we still do not understand their exact function, as their effects on epithelial barriers are inconsistent [30]. What we know, however, is that the importance of claudins in maintaining a normal physiologic state is clear and the dysregulation of their expression and/or interaction plays an important role in carcinogenesis. 

Claudins-3 and -4 are upregulated in a number of cancers, including ovarian, uterine, breast, prostate and pancreatic [9,31,32,33,34]. Additionally, claudin-3 overexpression is associated with increased malignant potential in colorectal cancer and breast cancer [35,36]. Claudin-3 and -4 have also been studied in uterine serous carcinoma, carcinosarcoma, and high-grade serous ovarian cancer, all of which are known for their aggressive behavior. Claudin-4 in particular is expressed in approximately 70%–90% of ovarian cancers and is differentially expressed across subtypes with the lowest expression seen in the clear cell subtype. In these tumors, differential expression of claudin-3 and -4 between cancerous and normal tissue suggest that these proteins may be exploited as either markers for early detection of carcinogenesis or as targets for tumor directed therapy [32,37,38,39,40]. 

### 2.2. Claudins as a Target of CPE

Since claudin-3 and -4 genes are highly and differentially expressed in biologically aggressive malignancies (including ovarian carcinoma), they represent a viable detection and treatment target [41]. Claudin-3 and -4 are natural receptors for *Clostridium perfringens* enterotoxin (CPE). The use of CPE for clinical benefit in claudin-3 and -4 expressing tumors has been evaluated. For example, in breast cancer, CPE-mediated toxicity was achieved by using claudin-3 and -4 as targets [42]. Furthermore, in prostate cancer, CPE-mediated cytotoxicity was identified when using claudin-4 as a target [43]. Lastly, claudin-4 was used as the target for CPE-mediated cytotoxicity in pancreatic cancer [44].

The expression of claudin-6 has been reported in multiple human cancers, including rhabdoid tumors, breast cancers and gastric cancers [45]. Claudin-6 is also expressed in ovarian cancer and may represent a novel functional receptor for CPE [10]. Interestingly, knock out claudin-6, as was done with UCI-101, an ovarian cancer cell line that is highly sensitive to CPE, decreased sensitivity to the toxin. Furthermore, when claudin-6 is overexpressed, different ovarian cell lines that are resistant to the effects of CPE become sensitive [10]. Lastly, binding assays reveal that CPE can bind claudin-6 in cells and this binding is associated with CPE cytotoxicity [10]. As a result of this research, claudin-6 has been established as a receptor for CPE and is now considered a therapeutic target in ovarian cancer and other cancers. 

### 2.3. Claudin-3 and -4 Are Potential Targets for CPE-Based Theranostics 

As natural receptors for CPE, claudin-3 and -4 are the members of the trans-membrane tissue-specific claudin family most capable of mediating CPE binding and cytolysis [12]. When the CPE toxin binds to cells, small pores form in the plasma membrane and osmotic equilibrium is lost. As a result, rapid cell death occurs [13]. The *C*-terminal fragment of CPE (*c*-CPE peptide) is composed of the CPE amino acids 184 to 319 with the receptor-binding region being in amino acids 290 to 319. *c*-CPE is a non-toxic ligand of claudin-3 and -4 and has fewer antigenic determinants. *c*-CPE can cause disruption of the tight junction barrier and thereby enhancing drug absorption through mucosal surfaces in a reversible and concentration-dependent manner [46]. A relatively low dose of *c*-CPE sensitizes epithelial ovarian cancer cells to the cytotoxic effects of carboplatin and paclitaxel in a claudin-4 dependent manner. Compared with single agent paclitaxel or carboplatin, the addition of *c*-CPE to paclitaxel is able to significantly suppress large tumor burdens by inhibiting tumor cell proliferation and accelerating apoptosis [47]. Furthermore, *c*-CPE can target TNFα to ovarian cancer cells and has been used as a carrier for other bacterial toxins aimed at claudin-4 positive tumor cells [48]. Yuan *et al.*, linked the *c*-CPE to TNFα. With *c*-CPE-TNF, they demonstrated 6.7-fold greater toxicity against the claudin-3 and -4 positive human ovarian carcinoma cells than free TNF, indicating that specific targeting to tumor cells expressing high levels of claudin-3 and -4 was achieved [48]. Unfortunately, thus far, this clinical application has been limited due to its failure to concentrate at the site of tumors and the associated side effects. 

Several research groups have reported using claudins-3 or -4 as targets for fluorescent molecule binding as a means to assist with the localization of ovarian and breast cancer cells [40,49]. *c*-CPE, when used as an intraperitoneal (i.p.) chemosensitizer, decreases tumor density and improves drug penetration by surface diffusion in ovarian cancer cells [50]*.* Since ovarian carcinoma is largely a disease of the peritoneal cavity and claudin-3 and -4 are not expressed in mesothelial cells, the utilization of i.p. treatment with full length CPE may have great potential for claudin-4 expressing ovarian tumors. Pharmacologic studies in ovarian cancer patients have demonstrated a therapeutic advantage to i.p. CPE and a significant reduction in systemic toxicity when compared with an identical dose of the drug given intravenously. Strategies to limit CPE toxicity to normal tissues already exist *in vivo* and are based on the local delivery of the blocking CPE peptide fragment to gut and lung via enteral and inhalation routes, respectively. These observations, combined with the finding that ovarian cancer remains confined to the peritoneal cavity for much of its natural history, suggest that i.p. administration of CPE in human patients harboring chemotherapy-resistant/residual disease may result in reduced toxicity and better therapeutic responses compared with identical doses of CPE given intravenously. Consistent with this view, multiple i.p. injections of sublethal doses of CPE every three days significantly inhibited tumor growth in 100% of mice harboring claudin-3 and -4 positive, chemotherapy resistant, ovarian tumor xenografts [23].

Other work in the area of chemoresistant ovarian cancer has demonstrated that CD44+ ovarian cancer stem cells represent a proportion of cancer cells capable of sustaining tumor growth and chemo-resistance. These cancer stem cells highly express genes encoding claudin-4. Casagrande *et al.* showed that multiple doses of i.p CPE were effective for the eradication of claudin-4 expressing chemoresistant ovarian cancer stem cells in mice harboring these xenografts. They showed a 100% reduction in tumor burden in half of the mice treated (*p* < 0.0001) [21]. In this study, recombinant CPE was not found to induce toxin-associated side effects. It should be noted, however, that repeated i.p. administrations were required in order to attain a therapeutic effect [51]. 

Another novel approach of safely targeting claudin-3 and -4 expressing ovarian tumor cells is through gene therapy. Intra-tumoral gene transfer of CPE-expressing vectors can be employed for selective suicide gene therapy of claudin-3 and -4 positive tumors. In one study, 72 hours after gene transfer, up to 100% cytotoxicity was noted in claudin-3 and -4 expressing tumor lines. The *in vivo* data from this study also revealed inhibition of ovarian cancer xenograft growth in SCID mice [52]. 

### 2.4. c-CPE-Based Diagnostic Agents in Gynecologic Cancers

Imaging of claudin-3 and -4 represents a promising diagnostic approach for identifying metastatic chemotherapy-resistant ovarian cancer [40]. When injected intravenously in mice harboring i.p. chemotherapy-resistant ovarian cancer, the non-toxic carboxy-terminal fragment of CPE (*c*-CPE) conjugated to the fluorescent FITC molecule revealed a preferential accumulation in the ovarian tumor when compared to the normal surrounding tissue. More importantly, the fluorescent *c*-CPE identified ovarian tumor spheroids isolated from the mouse ascites [40]. This research demonstrated that using a FITC-conjugated CPE peptide *in vitro* and *in vivo* revealed binding to multiple primary chemoresistant ovarian carcinoma cell lines and xenografts. This suggests that CPE peptide is a good candidate for tumor therapy and/or the development of new diagnostic tracers (*i.e*., radioisotopes), including the use of near-infrared fluorescent imaging to identify microscopic metastatic ovarian cancer preoperatively or at the time of tumor recurrence [40]. It also suggests that *c*-CPE can be complexed to biocompatible nanocarriers for the delivery of contrast agents (*i.e.*, iron oxide) or therapeutics (*i.e.*, chemotherapy, gene therapy) [8]. 

Another interesting approach is *c*-CPE conjugated to fluorescent dyes (*i.e.*, FITC or IRDye CW800). It has been employed as an intra-operative imaging system aimed at guiding surgeons to identify residual disease during real-time debulking surgery [40]. Real-time intra-operative approach imaging has recently been reported in humans undergoing surgery for ovarian cancer using folic acid conjugated to the FITC molecule [53]. The success of such a strategy resides on the fact that ovarian cancer metastases are commonly found attached to the organs of the peritoneal cavity and therefore easily exposed to the illumination source [54]. Uterine Serous Carcinoma (USC), a biologically aggressive variant of endometrial cancer, spreads in a pattern similar to high-grade ovarian cancer [55]. Furthermore, USC expresses high levels of claudin-3 and -4 and *in vitro* and *in vivo* is extremely sensitive to CPE [32]. This suggests that fluorescent *c*-CPE may be useful intra-operatively for the management of USC. Claudin-3 and -4 are also overexpressed in other solid tumors such as pancreatic, breast and prostate cancer [9,34,51,56]. The work of Kominsky *et al.* in particular showed that claudin-3 and -4 were consistently expressed in breast cancer brain metastasis, while negligibly expressed in healthy regions of the Central Nervous System (CNS) [51]. In line with this, intracranial administration of the full length CPE in mice harboring metastatic breast cancer in the brain significantly prolonged their survival compared to control animals. This suggests that *c*-CPE-based theranostic approaches may be useful for breast cancer brain metastasis identification. 

### 2.5. Future Directions

Currently, there are no early detection systems used to identify microscopic, recurrent and/or chemotherapy resistant gynecologic tumors. There are also no established or clinically effective treatment regimens available. Experimental results suggest that by using claudin-3 and -4 as targets and also as receptors for *c*-CPE conjugated to fluorescent dyes, toxins, and radioisotopes or combined with biodegradable/biocompatible nanoparticles for use in imaging, we will be able to diagnose and treat disease earlier and on orders of magnitude smaller than what is currently detected with imaging modalities today [40]. This novel approach is desperately needed to identify and ultimately achieve a cure for uterine and ovarian cancer that is recurrent and/or chemotherapy resistant.
